# Are preferences over health states informed?

**DOI:** 10.1186/s12955-017-0678-9

**Published:** 2017-05-18

**Authors:** M. Karimi, J. Brazier, S. Paisley

**Affiliations:** 1 Health Economics and Decision Science, School of Health and Related Research, University of Sheffield, Sheffield, UK; 2 Health Economics and Evidence Synthesis Research Unit, Luxembourg Institute of Health, Strassen, Luxembourg

**Keywords:** Informed preferences, EQ-5D, QALY, Health state valuation

## Abstract

**Background:**

The use of preference-elicitation tasks for valuing health states is well established, but little is known about whether these preferences are informed. Preferences may not be informed because individuals with little experience of ill health are asked to value health states. The use of uninformed preferences in cost-effectiveness can result in sub-optimal resource allocation. The aim of this study was to pilot a novel method to assess whether members of the public are informed about health states they value in preference-elicitation tasks.

**Methods:**

The general public was said to be informed if the expectations of the public about the effect of ill health on people’s lives were in agreement with the experience of patients. Sixty-two members of the public provided their expectations of the consequences of ill health on five life domains (activities, enjoyment, independence, relationships, and avoiding being a burden). A secondary dataset was used to measure patient experience on those five consequences.

**Results:**

There were differences between the expectations of the public and the experience of patients. For example, for all five life consequences the public underestimated the effects of problems in usual activities compared to problems in mobility. They also underestimated the effect of ‘anxiety or depression’ compared to physical problems on enjoyment of life and on the quality of personal relationships.

**Conclusions:**

This proof-of-concept study showed that it is possible to test whether preferences are informed. This study should be replicated using a larger sample. The findings suggest that preferences over health states in this sample are not fully informed because the participants do not have accurate expectations about the consequences of ill health. These uninformed preferences may not be adequate for allocation of public resources, and research is needed into methods to make them better informed.

## Background

Health economists have high expectations of members of the public. For example, in health state valuation tasks members of the public are asked to make complicated choices between health states that they may have never experienced [1]. The preferences elicited in these tasks are used in the calculation of QALYs and in cost-effectiveness analyses [1–3]. For example, the National Institute for Health and Care Excellence (NICE) in England recommends the use of a preferences-based tariff of the EQ-5D health instrument [4] in cost-effectiveness analysis. It is assumed that these preferences over health states are informed [1] (pp. 114–116) [2, 3], but the assumption may be difficult to justify considering the potential lack of experience of members of the public with the health states they are asked to value. If preferences over health states are not informed, the satisfaction of these preferences may not reflect improvements in welfare [5, 6] and resource allocation using those preferences may be sub-optimal. This means that researchers should focus on developing and testing methods to elicit more informed preferences.

Despite suggestions in the literature about the need for informed preferences, it is not clear what individuals should be informed about and how to test whether preferences are informed. Previous empirical work has shown that there is some evidence that members of the general public overestimate the effect of some health problems on subjective well-being (SWB) [7] and have difficulty assessing the effect of adaptation [8]. These studies have generally concluded that preference-based valuations value mental health dimensions less than SWB-based valuations [9, 10]. Yet, qualitative research has shown that when individuals value health states they consider factors beyond SWB [11–13]. The authors are not aware of a literature on whether members of the general public are informed about the wider consequences of the health states they are valuing.

To determine what individuals should be informed about, one must know how individuals evaluate health states. It has been theoretically argued that health is not valued for its own sake but is valued for its wider effect on an individual’s life [3, 14]. A previous qualitative study of health state valuation tasks based on this insight found that individuals value health states primarily by assessing the effect of ill health on six life consequences: activities, enjoyment, independence, relationships, dignity, and avoiding being a burden [13]. Independence and avoiding being a burden differ in that independence emphasises the effect of ill health on oneself while burden emphasises the effect on others, which may not always correlate. Thus, problems in the health dimension of ‘mobility’ (one of the dimensions of the EQ-5D health questionnaire) were valued for the effect they would have on a life consequence such as independence. These non-health consequences, such as enjoyment and dignity, are closer to quality of life dimensions than health dimensions [15] and the importance of non-health consequences has been a consistent finding in qualitative work on how individuals value health [12, 16]. The importance of the consequences suggests that ill health matters to the degree that it affects consequences.

The viewpoint of health being valued for its consequences has a justification in economic consumer choice theory. Lancaster [17] argued that an individual’s utility is based on the characteristics of a bundle of goods, rather than on the goods themselves. Likewise, a health state can be viewed as a good and the consequences of a health state can be viewed as its characteristics. The utility of a health state h, for time period t, has been expressed as U(h,t) [18], but the utility function can be also expressed as U(z):

$$ \mathrm{U}\left(\mathrm{z}\right)=\mathrm{f}\left({\mathrm{c}}_1\left(\mathrm{h},\mathrm{t}\right),\dots, {\mathrm{c}}_{\mathrm{j}}\left(\mathrm{h},\mathrm{t}\right)\right) $$

where c_1_…c_j_ are “consumption technology” functions (Lancaster [17]) that convert the health state and the time period into a set of characteristics or consequences, and f is a function to combine the characteristics. The “consumption technology” function can be assumed to be objective [17]. That is, a particular individual in a particular health state will obtain a particular characteristic and this characteristic is, in principle, objectively measurable. Thus, a particular individual with a particular type of depression is imposing a particular level of burden on others, and this level is not a matter of opinion, but of fact. Note that when measuring this level of burden it can be measured subjectively, for example, one individual can view a level of burden as ‘moderate’ whereas another individual may view that level of burden as ‘severe’. The “consumption technology” function is not necessarily universal, meaning that a health state can produce different characteristics for different individuals. Thus, two individuals with a particular type of depression can impose different levels of burden on others, perhaps because one individual has access to social services.

This characteristics approach to health states suggests that one aspect of having informed preferences over health states would be for individuals to have accurate knowledge about the consequences of ill health. The individual must first assess what consequences are important to him/her. Then, an individual must estimate his/her own consumption technology function and must determine what consequences he/she will obtain from that health state. The importance of each consequence is a value judgement, but the consequences of a health state are a matter of fact. The importance of independence for an individual is a value judgement, but the level of independence in a given health state is not.

Various reasons for the difference between patient and general public preferences have been suggested, which include adaptation and response shift, the possibility that patients and the general public have inherent differences in preferences, and inadequate descriptions of health states [1, 19]. In this paper the focus is on an alternative reason, namely whether individuals can correctly predict the consequences of ill health. If individuals are systematically mistaken about the consequences of ill health, their preferences over health states are not informed. The aim of this paper is to use the characteristics approach of health state valuation to pilot and demonstrate a novel test of informed preferences which can determine whether preferences over health states held by members of the general public are informed.

## Methods

### Overall study design

This study compares expectations of members of the general public about the consequences of ill health with the experience of patients, who are in some sub-optimal health state. First, the expectations were elicited of members of the general public concerning the effect of a set of EQ-5D-5L [20] health states on five of the consequences identified in a previous study by Karimi et al. [13] (activities, enjoyment, independence, relationships, and avoiding being a burden). Then, the experience of patients on those consequences was measured using a secondary dataset that contains self-reported data on patients’ health states and consequences. The sixth consequence (dignity) reported in Karimi et al. [13] was not used because no dataset was found that contained self-reported EQ-5D-5L and dignity data.

Regression models were estimated to establish the relationships between health and the five consequences based on patient experience. The results of the regressions were used to estimate the experience of patients for the same set of health states used in the expectations dataset. The estimates of the experience were used to rank the health states for each of the five consequences. The health states were also ranked using the expectations data from the general public. Finally, the expectations and the experience were compared. Because the experience and expectations use different measurement scales, only the ordinal placement of the health states was compared. No statistical significance testing could be undertaken because the ranking of the expectation dataset was based on only one data point (the mean). If members of the general public are informed, there should be no difference between the ranking of the expectations of the public and the experience of patients. The difference in the two rankings was expressed in the number of pairwise difference. A ranking of six health states suggests 15 unique orderings between each pair of the health states (i.e. the first ranked health state is ranked higher than the 5 below, the second health state is ranked higher than the 4 below, etc.). The two rankings are compared by counting how many of these unique orderings are different between the two rankings.

### Datasets used

Two sources of data were used in this study. The first data source is the data on expectations of members of the general public. The data was collected as part of a study where a convenience sample of members of the general public engaged in a reflection and deliberation exercise (for further details of the deliberation exercise see [21]). Participants were recruited by contacting University of Sheffield staff and students; by contacting voluntary, community, faith sector, and health or social care organisations in Sheffield [22]; and by using the snowball method. Recruiting was conducted using email and newsletter advertisements. Participants received £15 for participating in the group meeting.

Participants were split in several groups, but the task in this study was completed individually. Participants were asked to score six health states defined by the EQ-5D-5L on a visual analogue scale from 0 (worst) to 100 (best) for five consequences using a self-complete booklet (the booklet used to collect the data is available on request). The health states are shown in Table 1. The EQ-5D-5L was used because it is mentioned in the NICE methods guides [23] and may be adopted in the future. The six health states were chosen to include a range of severities and to contain deficits in different health dimensions. Each health state of the EQ-5D-5L can be described by a five digit number, with each digit representing the level of one dimension. For example, the number 13321 represents no problems on mobility and anxiety or depression, moderate problems on self-care and usual activities, and slight problems on pain or discomfort. The questions used to measure expectations are listed in Table 2. The questions were developed to closely resemble the consequences found in [13]. The dataset contains 62 respondents with no missing data.

**Table 1:** Health states used in this study

EQ-5D-5L health state	Value^a^	Description
11331	0.760	No problems in walking about; no problems washing or dressing myself; moderate problems doing my usual activities; moderate pain or discomfort; not anxious or depressed
31131	0.727	Moderate problems in walking about; no problems washing or dressing myself; no problems doing my usual activities; moderate pain or discomfort; not anxious or depressed
32322	0.573	Moderate problems in walking about; slight problems washing or dressing myself; moderate problems doing my usual activities; slight pain or discomfort; slightly anxious or depressed
11334	0.476	No problems in walking about; no problems washing or dressing myself; moderate problems doing my usual activities; moderate pain or discomfort; severely anxious or depressed
44535	−0.020	Severe problems in walking about; severe problems washing or dressing myself; unable to do my usual activities; moderate pain or discomfort; extremely anxious or depressed
44553	−0.118	Severe problems in walking about; severe problems washing or dressing myself; unable to do my usual activities; extreme pain or discomfort; moderately anxious or depressed

^a^Using the crosswalk UK value set [52]

**Table 2:** Phrasing, response options, and source of questions used for patient experience and public expectations data

Consequence	Expectations phrasing	Scale expectations	Source of experience question	Experience phrasing	Scale experience
Activities	Would you feel able to do the things and activities that you want to do?	100: Completely 0: Not at all	SF-36^a^	During the past 4 weeks, have you had any of the following problems with your work or other regular daily activities as a result of your PHYSICAL health? Cut down the amount of time you spent on work or other activities.	1: None of the time 2: A little of the time 3: Some of the time 4: Most of the time 5: All of the time
				(Same as above)…EMOTIONAL problems (such as feeling depressed or anxious)?	1: None of the time 2: A little of the time 3: Some of the time 4: Most of the time 5: All of the time
Relationships	Would you feel satisfied with your personal relationships	100: Completely 0: Not at all	ICECAP-A^b^	Love, friendship, and support	1: I can have a lot of love, friendship, and support 2: I can have quite a lot of love, friendship, and support 3: I can only have a little love, friendship, and support 4: I cannot have any love, friendship, and support
Independence	Would you feel independent and in control of your life	100: Completely 0: Not at all	ICECAP-A	Being independent	1: I am able to be completely independent 2: I am able to be independent in many things 3: I am only able to be independent in a few things 4: I am unable to be at all independent
Burden	Would you be able to avoid being a burden on others	100: Completely 0: Not at all	AQoL-8D^c^	How much of a burden do you feel you are to other people?	1: Not at all 2: A little 3: A moderate amount 4: A lot 5: Totally
Enjoyment	Would you feel you are able to enjoy life?	100: Completely 0: Completely unhappy	Integrated Household Survey^d^	Overall, how happy did you feel yesterday?	11: Completely happy to 0: Not at all happy

^a^ [26], ^b^ [44], ^c^ [53], ^d^ [54]

The second data source contains data on patients’ experience on the five consequences. The questions are listed in Table 2. These data were collected in the Multi Instrument Comparison (MIC) study [24], which was a cross-sectional international study conducted to compare measures of health and SWB. The MIC study was conducted online in six countries: Australia, UK, USA, Canada, Norway, and Germany. Respondents in the MIC study were members of an online panel and individuals with a self-reported disease diagnosis (79% of sample) and a demographically representative general public sample (21%). The respondents were first asked three subjective well-being questions [25]. The main questionnaire consisted of completing eight questionnaires: EQ-5D-5L, AQoL-8D, HUI-3, 15D, QWB-SA, SF-36, ICECAP-A, and a background questionnaire [24, 25]. A set of stringent data quality criteria were used [24, 25]. The total sample size for the MIC study is 8022.

Each consequence question in the public expectation dataset was compared with one from the patient experience dataset (see Table 2). One item measuring each consequence was selected from the questionnaires available in the MIC dataset. If multiple items were available, the item that was judged by the study team to be most closely conceptually related to the consequence and to the phrasing of the item in public expectation dataset was selected. For example, for the consequence of independence, the independence item from the ICECAP-A was chosen. The scale of the experience and expectations items differed: four to 11 response levels in the MIC data set but 0 to 100 in the public expectations data set. The activities question in the patient experience dataset was adjusted because, unlike the expectations question, the SF-36 question separated limitations caused by mental and physical health problems [26]. To obtain activity limitations due to both physical and mental health problems, the responses to the SF-36 question were adjusted by selecting the most severe reported response on the mental or the physical health item.

### Regression analyses and predictions

In the regression analyses the aim was to estimate the association between the consequences (dependent variable) and the EQ-5D-5L dimension levels (independent variables). The EQ-5D-5L contains 5 levels of 5 dimensions (with levels ranging from no problems to extreme problems/unable to) and was represented using 20 dummy variables. Four consequences in the patient experience data were treated as ordinal: activities (measured on 5 levels), independence (4 levels), relationships (4 levels), and burden (5 levels). The enjoyment consequence has 11 response levels but was treated as a continuous variable because Walters [27] recommends treating variables with seven or more response levels as a continuous measure and because it has been shown that assuming cardinality makes little difference to model estimates for enjoyment [28]. For ordinal variables a commonly used model is the cumulative logit model [27, 29]. This model makes the proportional odds assumption [27], which can be tested by comparing the cumulative logit model to a multinomial logistic model (MNL) using the chi squared score test [30–32]. In cases where the proportional odds assumption fails other models can be used. One alternative model is the stereotype logistic (STR) model [27], which does not make the proportional odds assumption but assumes the data is ordinal [29, 33, 34]. The STR model predictions can be compared to the MNL model, which relaxes the assumptions of the STR further [35](p. 282). If the predictions of the MNL and the STR are similar, the parsimonious nature of the STR would make it the preferred model [35](p. 282). Thus, if the proportional odds assumption was not valid and the predictions of the STR were similar to the MNL then the STR model was used.

A beta regression was used to analyse enjoyment because the data is bounded between 0 and 10 and an OLS is not appropriate for bounded data [36]. First, the enjoyment variable was transformed to a 0 to 1 scale. The beta regression cannot handle values of 0 and 1 and a transformation is applied to marginally compressed the values so that the model can be estimated [36]:

$$ {Y}^{\ast }=\frac{Y\left( N-1\right)+0.5}{N} $$

where Y* is the transformed dependent variable, *Y* is the 0 to 1 bounded transformed data, and N is the sample size.

No background variables were used in the regressions because it is not possible to know what background characteristics the public respondents were imagining when scoring the health states. Thus, the addition of background variables in the experience data regressions would not be useful.

It is possible that estimated regression coefficients are inconsistent, meaning worse health is associated with better consequences. This is, arguably, illogical. Therefore, for the EQ-5D dimensions with inconsistent and statistically insignificant coefficients the levels of the EQ-5D dimensions were combined until there were no longer any inconsistent coefficients.

After the regression models have been estimated they were used to estimate the experience of patients for the six EQ-5D health states for each consequence. For the ordinal models, probabilities of being in a response level for each health state were estimated. To get an overall expected value per health state the probabilities and response levels must be combined. This was done by multiplying the estimated probability by the response level and summing the total. The response levels were numbered from 1 to n, where n is the worst level. The total sum for each health state was used as a measure of the average experience in the health state. The beta regression model will produce a predicted value between 0 and 1 for each health state. This value is the measure of the patient experience for the enjoyment consequence.

All data analysis was done using R [37] with the VGAM package [38], the MASS package [39], and the betareg package [40]. Only complete cases with no missing data were used. A sensitivity analysis was conducted by using only UK-based respondents in the MIC dataset, but the conclusions of the paper did not change. Ethics approval was obtained for the use of secondary data and for the primary data collection from the School of Health Related Research at the University of Sheffield.

## Results

### Characteristics of samples

Socio-demographic characteristics for both samples are shown in Table 3. The median age of the general population expectations sample was 44 (1st quartile: 25, 3rd quartile: 62) and the median EQ-5D-5L value was 1 (0.77 and 1). About 59% of respondents were female. The median age in the patient experience data was 56 (42 and 66) and the median EQ-5D-5L value was 0.77 (0.66 and 0.88). About 52% of respondents were female. The distribution of the responses on the EQ-5D-5L from the patient experience sample are shown in Table 3. ‘No problems’ was the most frequently reported response for four of the five EQ-5D dimensions, while for pain or discomfort it was ‘slight’. The ‘unable to’/extreme level was infrequently reported, ranging from 0.4% in mobility to 2% in anxiety and depression. Median enjoyment was 0.7 (0.5 and 0.9). For the four other consequences the best response level was the most frequently reported.

**Table 3:** Background characteristics and distribution of health and consequences variables

	Patient experience dataset		Public expectations dataset		
Number of respondents	8022		62		
Median age (1st quartile, 3rd quartile)	56 (42, 66)		44 (25, 62)		
Median EQ-5D value (1st quartile, 3rd quartile)^a^	0.75 (0.66, 0.88)		1 (0.77, 1)		
Female, n (%)	4174 (52%)		37 (60%)		
Not married, n (%)	2883 (36%)		29 (47%)		
Degree, n (%)	2259 (28%)		41 (66%)		
Employed, n (%)	3685 (46%)		21 (34%)		
Retired, n (%)	2109 (26%)		17 (27%)		
Student, n (%)	323 (4%)		21 (34%)		
Individuals in EQ5D-5L domains for patient experience dataset regressions	Levels				
	1 (Best)	2	3	4	5 (worst)
Mobility, n (%)	5337 (66.5%)	1491 (18.6%)	824 (10.3%)	340 (4.2%)	30 (0.4%)
Self-care n (%)	7033 (87.7%)	646 (8.1%)	273 (3.4%)	62 (0.8%)	8 (0.1%)
Usual Activities n (%)	5182 (64.6%)	1739 (21.7%)	794 (9.9%)	256 (3.2%)	51 (0.6%)
Pain or Discomfort n (%)	2340 (29.2%)	3251 (40.5%)	1619 (20.2%)	697 (8.7%)	115 (1.4%)
Anxiety or Depression n (%)	4012 (50%)	2348 (29.3%)	1107 (13.8%)	393 (4.9%)	162 (2%)
Individuals in consequence levels for patient experience dataset regressions	Levels				
	1 (Best)	2	3	4	5 (worst)
Activities n (%)	3826 (47.7%)	1383 (17.2%)	1382 (17.2%)	827 (10.3%)	603 (7.5%)
Burden n (%)	4179 (52.1%)	2332 (29.1%)	894 (11.1%)	465 (5.8%)	152 (1.9%)
Independence n (%)	3601 (52.6%)	2521 (36.8%)	633 (9.2%)	90 (1.3%)	N/A
Relationships n (%)	2968 (43.4%)	2609 (38.1%)	1146 (16.7%)	122 (1.8%)	N/A
Median Enjoyment (1st quartile, 3rd quartile)	0.7 (0.5, 0.9)				

^a^Using the crosswalk UK value set [52]

### Regression results

All adjusted consistent models are reported in Table 4 (full models are available from the authors). A chi square test comparison of the ordered logit model with the multinomial model shows that the proportional odds assumption is violated for all four ordinal consequences. Correlations of predictions from the STR and MNL model were high (0.8 to 0.99). Therefore, the ordinal consequences were modelled using the STR model. For some consequences the adjusted consistent model indicates that many of the health dimensions in the EQ-5D were not associated with problems in the consequences. For example, when adjusting for an individual’s other health dimensions, having any mobility problems was associated with very little increase in personal relationship problems. Overall, the models pseudo R^2^ figures show that substantial variations of the consequences are unexplained by EQ-5D health states.

**Table 4:** Final model for five criteria. Reference category is the best outcome level. Estimates are log odds form

	Activities	Relationships	Independence	Burden	Enjoyment
Independent variables	Coefficient (SE)	Coefficient (SE)	Coefficient (SE)	Coefficient (SE)	Coefficient (SE)
Mobility level 2	0.63 (0.11)**	0.02 (0.04)**	0.43 (0.14)**	0.36 (0.14)**	0.05 (0.04)
Mobility level 3	0.73 (0.18)**		0.47 (0.19)**	0.5 (0.21)**	
Mobility level 4 or 5	0.89 (0.35)**		1.47 (0.32)**	1.71 (0.32)**	
Self-care level 2	0.39 (0.17)**	0.34 (0.02)*	1.36 (0.18)**	1 (0.18)**	−0.09 (0.05)*
Self-care level 3	1.37 (0.41)**	0.61 (0.33)**	2.41 (0.33)**	1.27 (0.31)**	
Self-care level 4 or 5	3.14 (0.97)**		3.82 (0.73)**	1.74 (0.63)**	
Usual activities level 2	1.74 (0.11)**	0.4 (0.1)**	1.14 (0.14)**	1.42 (0.15)**	−0.05 (0.04)
Usual activities level 3	3.44 (0.22)**		2.26 (0.23)**	2.3 (0.23)**	
Usual activities level 4 or 5	8.41 (0.66)**		4.26 (0.4)**	3.62 (0.36)**	−0.18 (0.08)**
Pain or discomfort level 2	0.52 (0.1)**	0.27 (0.04)*	0.11 (0.12)*	0.1 (0.13)*	−0.07 (0.03)**
Pain or discomfort level 3	0.78 (0.13)**			0.34 (0.17)**	−0.09 (0.04)**
Pain or discomfort level 4	1.39 (0.21)**			0.43 (0.23)*	−0.18 (0.06)*
Pain or discomfort level 5	2 (0.6)**			0.65 (0.46)*	
Anxiety or depression level 2	1.26 (0.09)**	2.65 (0.16)**	1.24 (0.12)**	2.16 (0.14)**	−0.78 (0.03)**
Anxiety or depression level 3	2.21 (0.12)**	4.41 (0.2)**	1.68 (0.16)**	3.93 (0.2)**	−1.45 (0.04)**
Anxiety or depression level 4	3.39 (0.23)**	5.89 (0.24)**	2.62 (0.25)**	5.88 (0.28)**	−2.22 (0.07)**
Anxiety or depression level 5	4.79 (0.44)**	6.99 (0.16)**	3.22 (0.38)**	7.51 (0.48)**	−2.85 (0.09)**
Theta 5	1	-	-	1	-
Theta 4	0.89	1	1	0.86	-
Theta 3	0.71	0.65	0.85	0.65	-
Theta 2	0.48	0.26	0.5	0.38	-
Theta 1	0	0	0	0	-
Constant 4	−4.99	-	-	7.69	-
Constant 3	−3.9	−6.66	−6.65	−5.43	-
Constant 2	−2.55	−2.69	−3.82	−3.43	-
Constant 1	−1.84	−0.6	−1.17	−1.38	-
Observations	8021	6845	6845	8022	8008
Residual deviance	18,113.5	13,419.76	11,273.81	15,451.22	-
Pseudo R^2^	0.18^a^	0.11^a^	0.16^a^	0.18^a^	0.21^b^
AIC^c^	18,162	13,448	11,312	15,499	−8388
Correct predictive ability based on in sample prediction	51%	52%	63%	58%	N/A

** *P*-value <0.05, * *P*-value <0.1; ^a^McFadden (adjusted); ^b^Square of the sample correlation coefficient between the outcome and predicted values, ^c^AIC Akaike information criterion; The base level chosen for the five EQ-5D dimensions and the five consequences variables is no problems

The anxiety or depression dimension had the highest odds ratio for relationships, burden, and the enjoyment consequences, while usual activities dimension had the highest odds ratio for the activities and independence consequences. Problems in these two dimensions tended to have the largest odds associated with reporting problems on the five consequences.

### Comparing the six health states using experience and expectations

**Fig. 1:**
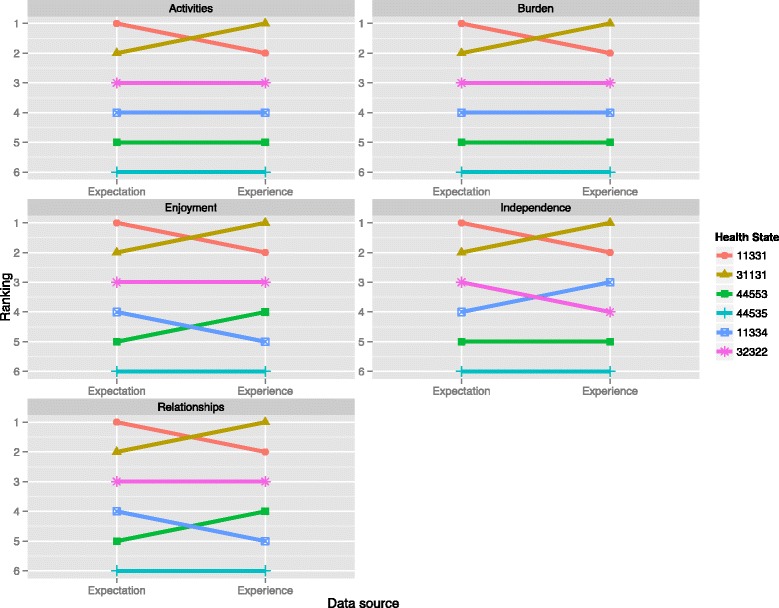
Comparison of expectations and experience rankings

The rankings of the health states according to both experience and expectations are shown in Fig. 1 (full probability predictions are available from the authors). According to the patient experience of all five consequences, health states 31131 and 11331 are ranked as first and second, while health state 44535 is ranked last. The rankings of the three health states 11334, 32322, and 44553 differ between the consequences. There were thus similarities but also differences in the ranking of the six health states across the five consequences in the patient experience dataset. In the public’s expectations data, the ranking was the same for all five consequences. The health states were ranked from best to worst as follows: 11331, 31131, 32322, 11334, 44553, and 44535.

Each consequence had at least one pairwise difference between the expectations of the public and the experience of patients. This resulted in a total of eight differences. Some of these differences were repeated across the consequences, five of the differences were between health states 11331 and 31131, two were between 11334 and 44553, and one was between 11334 and 32322. The difference in ranking between health states 11331 and 31131 occurred in all consequences. For each consequence, the public expected moderate mobility problems to be worse than moderate problems with usual activities, but moderate problems in usual activities was associated with worse consequences than mobility problems.

The difference in ranking between health states 11334 and 44553 was found in relationships and enjoyment. The public expected health state 44553 to be worse than 11334, but the patient experience of health state 11334 was associated with worse consequences. A one level detriment in anxiety or depression was thus associated with worse personal relationships and enjoyment than the additional problems in the other four dimensions. The difference in ranking between health state 11334 and 32322 was found in the independence consequence. The public expected health state 11334 to be worse than state 32322, but health state 32322 was associated with worse independence according to the experience data. Therefore, the public overestimated problems in anxiety or depression and pain or discomfort compared to problems in self-care and mobility for the independence consequence.

## Discussion

Overall, eight differences were found between the rankings of health states using the expectation of the public and the experience of patients. Given that there were six health states and five consequences, a maximum of 75 (15 per consequence) pairwise differences could have been found. Therefore, 11% of the total possible pairwise differences were found. This figure is an understatement of the extent of differences between expectations and experience because from the 15 potential pairwise differences per consequence, a total of eight pairwise comparisons involve a health state that dominates the other (i.e. in the pairwise comparison a health state has either equal or less problems in every dimension than the other health state). The eight differences therefore represent 23% of total non-dominated pairwise comparisons (8/(7*5)). The findings in this paper may be a conservative estimate of the true discrepancy between expectations and experience because the comparison was at an ordinal level and thus cardinal differences could not be investigated. Overall, this study indicates that this novel method can be used to assess whether members of the public are informed and the evidence suggests that although the participants in this study are not grossly misinformed about how health affects the six consequences, their expectations are not accurate even on an ordinal scale.

The most frequent difference in the ranking was because the public underestimated the effects of moderate problems in usual activities compared to moderate problems in mobility. Both other differences in ranking involved health state 11334. For the consequences of enjoyment and relationships health state 11334 was underestimated compared to health state 44553. This meant that severe anxiety or depression was underestimated compared to problems in the other four dimensions. For the consequence of independence, health state 11334 was overestimated compared to 32322. This means that anxiety or depression combined with pain or discomfort was overestimated compared to problems in mobility and self-care for independence.

The STR and beta regression results for patient experience indicate that anxiety or depression and usual activities are the two dimensions with the largest odds ratios for the five consequences. These results have face validity given that some consequences would be expected to correlate with some health dimensions. For example, for the activities consequence it was the usual activities dimension that was associated with the largest odds ratio and for enjoyment it was the anxiety or depression dimension that was associated with the largest odds ratio.

The regression results of this study confirm the findings in the literature that the association of the mental health dimension (i.e. anxiety or depression) with subjective well-being is stronger than with other health dimensions [9, 10, 41]. Dolan and Metcalfe [9] find that problems in anxiety or depression are associated with a detriment in enjoyment about 10 times as large as mobility problems. Similarly, in this study the beta regression model estimated that odds of having worse enjoyment were higher for anxiety or depression problems than for any other dimension. Dolan and Metcalfe [9] argue that comparing values derived from preferences to measurements of subjective well-being shows that members of the general public undervalue mental health compared to physical health. In this study, the public underestimated the effect of having anxiety or depression problems on enjoyment and personal relationships when comparing states 11334 to 44553. However, in this study anxiety or depression was not underestimated when comparing states 44535 to 44553 (i.e. comparing extreme pain or discomfort to extreme anxiety or depression directly). No literature has been identified on whether anxiety or depression is underestimated for other consequences. While in this sample the public underestimated the effect of anxiety or depression on enjoyment and relationships, they did not underestimate the effect of anxiety or depression on other consequences.

The findings of this study, if replicated in a larger study, have implications for the use of preference-elicitation tasks for resource allocation in health care. For example, the public’s beliefs about the consequences of problems in usual activities compared to problems in mobility were not in line with patient experience. This can mean that the use of preferences for evaluating interventions undervalues improvements in usual activities compared to improvements mobility, although the problem is lessened if improvements in the two dimensions are correlated. Similarly, for consequences such as enjoyment and relationships the public underestimated problems with anxiety or depression compared to problems in the physical dimensions. As a result, interventions that improve mental health could be undervalued. Using uninformed preferences to value the EQ-5D could thus result in sub-optimal policy recommendations. Research that focuses on encouraging more informed preferences by developing methods to inform the public of the consequences of health states could be continued [42]. One possibility for better informed preferences is to provide members of the general public with more information about the experience of patients. There is also the possibility to move further away from existing methods. One suggestion in the literature is the use of patient preferences, which may have the benefit of more closely matching experience and expectations, but requires patients to imagine full health [8] and has practical limitations [43]. An altogether different approach is to use general population preferences by developing a descriptive classification based on the consequences, perhaps by using ICECAP-A [44] or another well-being based descriptive system [43]. In addition, it would be important to know how different informed and uninformed preferences are, and if those differences are of practical significance to cost-effectiveness analysis. This research will ultimately result in a value set that is more defensible and is more in line with what the general public would want if they were informed about the consequences of health states.

The limitations in this study include the phrasing of the expectations and experience questions, the method of comparing the two datasets, and the study sample. As shown in Table 2, there are differences in the phrasing of the questions in the participant expectations and patient experience datasets. For example, the experience independence question (originally in the ICECAP-A [44]) does not mention control but the expectation question does. Another difference between the expectations and experience is that the scales are different, and therefore comparison could only be made on an ordinal basis. It may be that ordinal rankings are correct, while relative cardinal values are not. The datasets used in this study have limitations. The MIC datasets had few respondents in the worst response level of the EQ-5D-5L dimensions and more observations for those levels would make inferences more reliable. The MIC datasets is cross-sectional and there is a potential for endogeneity in this type of cross-sectional datasets [45]. A longitudinal panel dataset would be useful to account for individual heterogeneity and can more easily assess causality [45]. Additionally, only six health states were used in this study. To obtain a broader view of the difference between expectations and experience a wider range of health states would be needed. This is further discussed in the future research section.

There are limitations in the sampling of both the public expectations and patient experience datasets. Ideally, both samples should be comparable and representative of the population. The sample of members of the general public in the expectations dataset was small and not representative of the general UK population. This is particularly important because average beliefs of members of the public were compared to average patient experiences. The MIC sample was an online sample, and although online samples are being used more frequently in health economics studies [46] they may suffer from self-selection (though any sampling method would). Lastly, this paper assumes that the ranking of the average of the public’s expectation should be the same as the ranking of the average patient’s experience. This assumption can be justified because the EQ-5D is a generic and broad instrument and various diseases map on to the same EQ-5D health profile. The average expectations thus should be close to the average experience. Furthermore, difference between expectations and experience could be caused by adaptation. The expectations of the general public are elicited by asking them to consider living 10 years in a health state, although they may rather be focused on the transition to ill health [7], but how long a patient has been in the health state is unknown. The effect of this is difficult to estimate, but it is not necessarily expected to affect the ordinal ranking of the experience and expectations of the health state.

Future research can, in the first instance, focus on addressing the limitations of this study to more accurately assess the extent to which preferences are informed. First, the sample could be improved by measuring expectations from a larger and representative group of members of the general public as is typically recommended for valuation studies [2]. Second, a larger number of health states could be included, which can provide a better overall indication of whether the public is informed. Third, the study methods could be improved by using the same question and scales for both the expected and experience questions, which would allow for better comparison between the two. If the same questions are used alongside a larger number of health states the analysis could then focus on analyzing the difference between expectations and experiences in a joint regression model and could thus include both statistical significance testing and could investigate health state dimensions and levels rather than health states, which may reveal whether there are certain dimensions about which the public is less informed.

One issue to be further investigated is the effect of using of self-reported questions to measure consequences and whether differences between experiences and expectations are partly driven by response scale heterogeneity [47]. In this study, one item from existing questionnaires to measure consequence was chosen, but other items that measure experience could be tested. There may also be a benefit to more sophisticated elicitation procedures. For example, probabilistic expectations can be elicited, which has been previously implemented to elicit expectations of future earnings given educational attainment [48]. In addition, uncertainty in beliefs may need to be included [48]. Different probability elicitation procedures exist for eliciting uncertainty and they may have implications for the truthfulness of reported expectations [49]. The method in this study could also be conducted with other generic preference-based measures such as the SF-6D [50] and HUI [51]. In particular, the comparison between measures based on the ‘within the skin’ approach and measures that focus more the social context or impairment may be interesting. It may be that judging the consequences is difficult when using a within the skin type measures, such as the HUI, and the results of this study cannot necessarily be generalized to other measures.

## Conclusion

This study has developed and implemented a novel method to determine whether preferences over health states are informed. It has shown that this method is feasible, could be implemented in a larger study, and can present important new knowledge on whether preferences are informed. The expectations of the public, in this study, did not always conform to the experience of patients, most consistently for the comparison between the EQ-5D-5L dimensions of usual activities and mobility problems and between anxiety or depression and other dimensions. This means that preferences of the study sample are not entirely informed, and preference-elicitation tasks with this sample may rest on false beliefs. QALY calculations based on those uninformed preferences may provide sub-optimal recommendations in cost-effectiveness analysis. This was a pilot study with limitations, but the findings indicate that a fuller investigation with a larger representative sample and more health states is warranted to assess whether members of the public are informed.

## Abbreviations

MIC: Multi instrument comparison study
NICE: National Institute for health and care excellence
QALY: Quality adjusted life year
STR: Stereotype regression
SWB: Subjective well-being
